# Cardiac diffusion kurtosis imaging in the human heart in vivo using 300 mT/m gradients

**DOI:** 10.1002/mrm.30626

**Published:** 2025-07-03

**Authors:** Maryam Afzali, Sam Coveney, Lars Mueller, Sarah Jones, Fabrizio Fasano, C. John Evans, Irvin Teh, Erica Dall'Armellina, Filip Szczepankiewicz, Derek K. Jones, Jürgen E. Schneider

**Affiliations:** ^1^ Biomedical Imaging Science Department, Leeds Institute of Cardiovascular and Metabolic Medicine University of Leeds Leeds UK; ^2^ Cardiff University Brain Research Imaging Centre (CUBRIC), School of Psychology Cardiff University Cardiff UK; ^3^ Siemens Healthcare Ltd. Camberly UK; ^4^ Siemens Healthcare GmbH Erlangen Germany; ^5^ Medical Radiation Physics, Clinical Sciences Lund Lund University Lund Sweden

**Keywords:** cardiac diffusion MRI, diffusion kurtosis imaging, strong gradients

## Abstract

**Purpose:**

Diffusion tensor imaging (DTI) is commonly used in cardiac diffusion magnetic resonance imaging (dMRI). However, the tissue's microstructure (cells, membranes, etc.) restricts the movement of the water molecules, making the spin displacements deviate from Gaussian behavior. This effect may be observed with diffusion kurtosis imaging (DKI) using sufficiently high b‐values ($b>450\;{\text{s/mm}}^{2}$), which are presently outside the realm of routine cardiac dMRI due to the limited gradient strength of clinical scanners. The Connectom scanner with $G_{\max}=300\;\mathrm{mT}/m$ enables high b‐values at echo times (TE) similar to DTI on standard clinical scanners, therefore facilitating cardiac DKI in humans.

**Methods:**

Cardiac‐gated, second‐order motion‐compensated dMRI was performed with $b_{\max}=1350\;s/mm^{2}$ in 10 healthy volunteers on a 3T MRI scanner with $G_{\max}=300\;\mathrm{mT}/m$. The signal was fitted to a cumulant expansion up to and including the kurtosis term, and diffusion metrics such as fractional anisotropy (FA), mean diffusivity (MD), mean kurtosis (MK), axial kurtosis (AK), and radial kurtosis (RK) were calculated.

**Results:**

We demonstrate deviation of the signal from monoexponential decay for b‐values $>450\;s/mm^{2}$ (MK=0.32±0.03). Radial kurtosis (RK=0.35±0.04) was observed slightly larger than axial kurtosis (AK=0.27±0.02), and the difference is statistically significant (RK−AK=0.08±0.04, p=2e−4).

**Conclusion:**

This work demonstrates the feasibility of quantifying kurtosis effect in the human heart in vivo (at an echo time shorter than typical TEs reported for cardiac DTI), using high‐performance gradient systems (which are 4–8 times stronger than on standard clinical scanners). Our work lays the foundation for exploring new biomarkers in cardiac dMRI beyond DTI.

## 1 INTRODUCTION

Diffusion magnetic resonance imaging (dMRI) is a non‐invasive technique to study tissue microstructure.^1^ So far, diffusion tensor imaging (DTI), which is obtained by combining dMRI measurements along at least six non‐collinear encoding directions, has been used most commonly in microstructural investigations of the heart.^2^

DTI is based on the assumption that the probability of finding a particle in position r at time t adheres to a Gaussian distribution. The standard deviation of this distribution is directly related to the diffusion coefficient. While cardiac diffusion tensor imaging (cDTI) can characterize the average displacement of water molecules in the three‐dimensional space, it is not able to provide more specific information about the underlying micro‐environment. This is a consequence of the Gaussian assumption in the DTI signal representation.^3^ dMRI signal is usually measured using diffusion‐sensitized sequences that vary the diffusion weightings (*b‐values*), which are influenced by both the gradient strength and the diffusion time. This signal behavior can be represented using a mono‐exponential decay, as described by the Stejskal–Tanner equation.^4^ However, due to cell membranes and other restrictions in biological tissue, the diffusion of water molecules deviates from Gaussian behavior.^5^, ^6^, ^7^ Consequently, the diffusion‐weighted signal in tissue deviates from monoexponential decay at higher b‐values as shown for human brain,^8^, ^9^ lung,^10^ prostate,^11^ breast,^12^ calf muscle,^13^ and liver.^14^ However, this has not been widely studied in the human heart in vivo so far.

The DTI representation results from truncating the cumulant expansion of the logarithm of the dMRI signal at the first term, where the logarithm of the signal is a linear function of b‐value. Conversely, the diffusion kurtosis imaging (DKI) representation truncates at the second term, such that the logarithm of the signal is quadratic in b‐value. Diffusional kurtosis is a measure of the restriction of water molecule movement, which in biological tissue is most likely attributable to cell membranes, organelles, and tissue compartments, among other factors.^15^ The ability to quantify restriction provides additional microstructural information beyond what is available from the diffusion coefficient alone, making it one of the key advantages of kurtosis imaging.^6^ Tissue structure and other factors, such as the concentration of macromolecules, impact the diffusion coefficient. The diffusion coefficient is therefore a less specific indicator of a tissue's structural complexity.^6^

The same type of pulse sequence employed for cardiac diffusion tensor imaging, can be used for DKI, but the required b‐values are larger than those usually used to measure diffusion coefficients ($b_{\max}=1500\;s/mm^{2}$ ^16^). The required b‐values can only be obtained at echo times comparable to those used on clinical MR scanners for conventional cDTI^17^, ^18^ if ultra‐strong gradient systems are available / used. The myocardium exhibits significantly shorter $T_{2}$ relaxation times compared to tissues where DTI and DKI are more commonly applied, such as the brain. Consequently, the ability to achieve shorter echo times is paramount for cardiac diffusion imaging. This necessity arises from the need to acquire sufficient signal before $T_{2}$ decay substantially decreases the measurable diffusion‐weighted signal, thereby enabling accurate and reliable diffusion and kurtosis measurements in cardiac tissue.

Non‐Gaussian diffusion in the myocardium has been investigated in preclinical experiments such as perfused rat, rabbit, guinea pig, and porcine hearts.^7^, ^19^, ^20^, ^21^, ^22^, ^23^ McClymont et al.^7^ demonstrated non‐Gaussian diffusion in healthy and hypertrophic rat hearts and showed that diffusion kurtosis along the second and third eigenvectors of the diffusion tensor can differentiate hypertrophic hearts from sham hearts. Although all of these studies indicate the potential of DKI to provide novel and more refined biomarkers of heart disease, the analysis of diffusion kurtosis in the human heart in vivo has been limited.

Teh et al.^16^ have recently demonstrated the feasibility to quantify non‐Gaussian diffusion in healthy volunteers using a conventional 3T MR system, albeit at long echo times (TE) (>100ms) and consequently, low SNR. Furthermore, the reported isotropic, anisotropic, and total kurtosis was directionally averaged and not directionally resolved in three dimensions.

Here, we investigate three‐dimensional diffusion kurtosis in healthy human hearts in vivo using ultra‐strong gradients (i.e., 300 mT/m)^17^, ^18^ at a TE = 61 ms—similar to the echo times commonly used for conventional cardiac DTI—and $b_{\max}=1350\;s/mm^{2}$ with a second‐order motion‐compensated waveform. To the best of our knowledge, this is the first in vivo quantification of three‐dimensional diffusion kurtosis in the human heart.

## 2 THEORY

The apparent kurtosis coefficient for a single direction can be determined by acquiring data at three or more b‐values and fitting the signal (S(b)) to the equation:

(1) $$\ln S(b)=\ln S(0)-bD_{\text{app}}+\frac{1}{6}b^{2}D_{\text{app}}^{2}K_{\text{app}}$$

where $D_{\mathrm{app}}$ is the apparent diffusion coefficient (ADC) for the given diffusion encoding axis, and $K_{\mathrm{app}}$ is the apparent kurtosis coefficient, a dimensionless parameter.

The non‐Gaussian behavior of water diffusion in the three‐dimensional space can be characterized by a symmetric 3×3×3×3 tensor, called the *kurtosis tensor*, W,

$$\begin{array}{ll} \ln\frac{S(n,b)}{S_{0}} & =-b\overset{3}{\underset{i=1}{\sum }}\overset{3}{\underset{j=1}{\sum }}n_{i}n_{j}D_{ij}\\ & \;+\frac{1}{6}b^{2}{\text{MD}}^{2}\overset{3}{\underset{i=1}{\sum }}\overset{3}{\underset{j=1}{\sum }}\overset{3}{\underset{k=1}{\sum }}\overset{3}{\underset{l=1}{\sum }}n_{i}n_{j}n_{k}n_{l}W_{ijkl} \end{array}$$

where **n** is the diffusion encoding vector, i, j, k, and l are indices of the directions in the physical space, and can take values of 1, 2, or 3. Because of the full symmetry of the tensor, only the following 15 elements^6^ are independent: $W_{1111}$, $W_{2222}$, $W_{3333}$, $W_{1112}$, $W_{1113}$, $W_{1222}$, $W_{2223}$, $W_{1333}$, $W_{2333}$, $W_{1122}$, $W_{1133}$, $W_{2233}$, $W_{1123}$, $W_{1223}$, $W_{1233}$.

$K_{\mathrm{app}}$ for the direction $n=(n_{1},n_{2},n_{3})$ can be calculated from $W_{\mathrm{ijkl}}$:

(2) $$K_{\text{app}}=\frac{{\text{MD}}^{2}}{D_{\text{app}}^{2}}\overset{3}{\underset{i=1}{\sum }}\overset{3}{\underset{j=1}{\sum }}\overset{3}{\underset{k=1}{\sum }}\overset{3}{\underset{l=1}{\sum }}n_{i}n_{j}n_{k}n_{l}W_{ijkl},$$

where

(3) $$\text{MD}=\frac{1}{3}\overset{3}{\underset{i=1}{\sum }}D_{ii}.$$

MD is the mean diffusivity and is independent of the direction, and $n_{i}$ is the component of the direction unit vector. For DTI, at least 7 measurements with one non‐zero b‐value are required to quantify the diffusion tensor (6 unique elements due to symmetry) and the non‐diffusion‐weighted signal. We need at least two non‐zero b‐values and 15+6+1=22 measurements to quantify the diffusion tensor D (6 unknowns), kurtosis tensor, W (15 unknowns), and non‐diffusion‐weighted signal ($S_{0}$). Various diffusion and kurtosis parameters can be calculated from D and W,^24^, ^25^, ^26^ where fractional anisotropy (FA), mean diffusivity (MD), mean kurtosis (MK), axial and radial kurtosis (AK and RK) are among the most widely used DKI parameters.^24^, ^27^, ^28^

## 3 METHODS

### 3.1 Experimental setup and recruitment

Cardiac diffusion‐weighted images (cDWI) were acquired on a Connectom 3T research‐only MR imaging system (Siemens Healthcare, Erlangen, Germany) with a maximum gradient strength of 300 mT/m and slew rate of 200 T/m/s. An 18‐channel body receive coil was used in combination with a 32‐channel spine receive coil. Ten healthy volunteers (with no known previous cardiac conditions) were recruited for this study (age range 20.1±1.6 years (18–22 years), weight range of 64±12 kg (54–94 kg), six females). The study was approved by the local institutional review board, and all subjects provided written consent.

### 3.2 Data acquisition

Routine GRE^29^ and TRUEFISP^30^ sequences were used for cardiac planning and cine‐imaging, whereas cDWI was performed with a prototype pulse sequence that enabled diffusion encoding with user‐defined gradient waveforms.^31^, ^32^ The cine images were acquired in short‐axis orientation for apical, mid, and basal slices. cDWI was performed at the same location and orientation as the cine imaging. The phase encoding direction was systematically varied in scout DW images (in steps of 30°), and the orientation providing the best visual image quality was chosen for the full cDWI acquisition in each subject.

Diffusion gradient waveforms were designed using the NOW toolbox^33^, ^34^ (https://github.com/jsjol/NOW) to provide second‐order motion‐compensated waveforms that can reach a specific b‐value in the shortest echo time. The maximum gradient strength used in this study for M2‐compensation acquisition to generate the b‐value of $1350\;s/mm^{2}$ was 285.4 mT/m and the maximum slew‐rate was 76.2 T/m/s (slew‐rate is limited due to peripheral nerve stimulation and cardiac stimulation limits, see^17^ for more details) which resulted in an echo time of 61 ms (Figure S1, Supporting Information). Thus, the waveforms here used the physiologically‐limited slew rate of ∼76.2T/m/s instead of the 200 T/m/s hardware limit. This added 6 ms to the echo time. In addition, diffusion gradient waveforms were designed for maximum gradient strengths of 200 and 80 mT/m corresponding to the Cima.X, and Prisma Siemens MRI scanners, respectively (Figure S1, Supporting Information).

The cDWI parameters were: TR = 3 RR‐intervals, field‐of‐view = $320\times 120\;mm^{2}$ using ZOnally‐magnified Oblique Multislice (ZOOM, tilted RF: Excitation, tilt angle: 15°, tilted slice thickness: 20 mm),^32^, ^35^ in‐plane resolution = $2.7\times 2.7\;mm^{2}$, slice thickness = 8 mm, 3 short axis slices (base, mid, and apical), partial Fourier factor = 7/8, no parallel imaging, bandwidth = 2354 Hz/pixel. Each full data set was comprised of 5 b‐values [b = 100, 450, 900, 1200, 1350 $s/mm^{2}$] in 30 directions per shell with 6 repetitions, except for the lowest b‐value which only had 3 directions and 12 repeats. Data were acquired with ECG‐gating and under free‐breathing.^2^ Saturation bands were placed around the heart to further suppress the signal from outside the volume of interest. Fat suppression was performed using the SPAIR method.^36^ The trigger delay was adjusted for cDWI acquisition in mid‐end systole as determined from the cine images. The total acquisition time was around one hour, where the nominal scan time of the DTI/DKI protocol was 40 min at 60 beats/min heart rate. Both magnitude and phase data were collected and used to generate complex‐valued images.

### 3.3 Data analysis

The phase variation in each complex‐valued diffusion‐weighted image was removed using the method proposed by Eichner et al.^37^ An in‐house developed toolbox was used for further post‐processing.^38^, ^39^ Real‐valued diffusion‐weighted images were first registered: For each slice, all low b‐value images were registered to one user‐specified low b‐value image, and then all images were registered to the mean of the co‐registered low b‐value images. The 2D registration was performed with SimpleITK,^40^ with rigid transformation, separately for basal, middle, and apical slices. Next, an outlier rejection technique was used to semi‐automatically remove the outliers (e.g., the images with misregistration or motion corruption) from the data.^38^, ^41^

The noise level, σ, was measured as the standard deviation of the real part of the noise data (acquired without RF pulses) in the image domain from 256 repetitions. The SNR of the data is defined as SNR = S/σ, where S is the average of measured signal intensity over the repeats at different b‐values in each voxel.^42^ To show the SNR values quantitatively for all subjects, we calculated the SNR per voxel for each b‐value and diffusion encoding direction (30 directions for $b>100s/mm^{2}$ and 3 directions for b = 100 $s/mm^{2}$), then the SNR values were averaged over different directions for each b‐value. SNR was calculated after image registration and before outlier rejection.

The diffusion tensor was fitted to four subsets of data including b = 100 $s/mm^{2}$ combined with different maximum b‐values (b = 100 and 450 $s/mm^{2}$, b = 100 and 900 $s/mm^{2}$, b = 100 and 1200 $s/mm^{2}$, b = 100 and 1350 $s/mm^{2}$) using weighted linear least squares regression (WLS),^43^ and diffusion metrics such as fractional anisotropy (FA) and mean diffusivity (MD) were calculated for each subset.

In addition, two subsets of data (subset 1: b = 100, 450, 900 $s/mm^{2}$; subset 2: b = 100, 450, 900, 1200 $s/mm^{2}$) were compared to the full set: b = 100, 450, 900, 1200, 1350 $s/mm^{2}$) for fitting both DTI and DKI.

The left ventricular myocardium was segmented manually in each slice.^38^ Areas corrupted by susceptibility‐related distortion—typically located between myocardium, deoxygenated blood, and air, particularly around the posterior vein—were excluded from calculating the global metrics.

Bland–Altman plots were used to compare the mean FA and MD obtained from different subsets of data. To determine the statistical significance between parameters, the Wilcoxon signed‐rank test was used; a p‐value less than or equal to 0.05 was considered statistically significant.

The DKI representation was then fitted to the data (all b‐values) using WLS and the diffusion metrics such as FA, MD, helix angle (HA), secondary eigenvector angle (E2A), mean kurtosis (MK), axial (AK), and radial kurtosis (RK) were calculated.^44^

Radial kurtosis (RK) and axial kurtosis (AK) were also compared using the Wilcoxon signed‐rank test.

## 4 RESULTS

**FIGURE 1:**
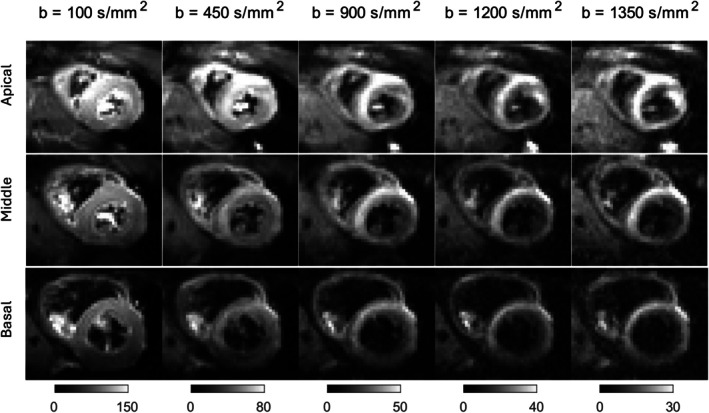
Example representative cardiac diffusion‐weighted images averaged over six repeats of a single diffusion direction acquired in basal, middle, and apical slices with b‐value = 100, 450, 900, 1200 and 1350 $s/mm^{2}$ using second‐order motion compensation with TE=61ms.

Figure 1 shows representative diffusion‐weighted images averaged over six repeats of a single diffusion direction acquired with b = 100, 450, 900, 1200, and 1350 $s/mm^{2}$ in short axis view. We calculated the number of rejected images at each b‐value and averaged it over 10 subjects. For b = 100, 450, 900, 1200, and 1350 $\left(s/mm^{2}\right)$, the average number of rejected images was 1.14, 3.86, 1.14, 3.14, and 1.14, respectively. These results indicate that the majority of b = 1350 $s/mm^{2}$ images were retained for DKI fitting. The rejected images were likely due to subject motion, which can occur at any b‐value during acquisition. This explains why the number of rejected images at b = 450 s/mm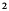 is higher than at b = 1350 $s/mm^{2}$, despite the latter having a higher b‐value and therefore lower SNR.

On average 12%±6% of the voxels in the left ventricular mask were discarded due to poor image quality and signal dropout.

The mean and standard deviation of the SNR at b = 100, 450, 900, 1200, and 1350 $s/mm^{2}$ were 40±10,23±6,12±3,9±2,7±2, respectively.

**FIGURE 2:**
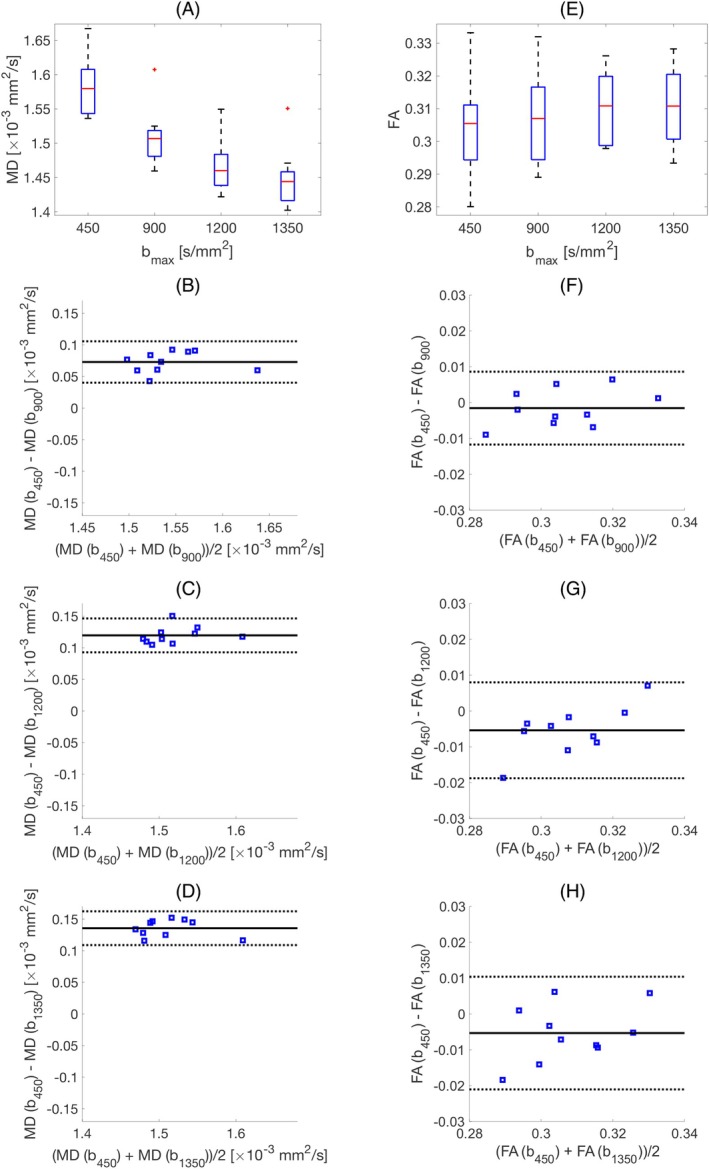
Change of MD and FA for different $b_{\max}$ values. (A) and (E) shows the box plot of mean MD and FA over 10 subjects (the red line shows the mean value). (B–D) and (F–H) Bland–Altman plots, comparing mean diffusivity (MD), and fractional anisotropy (FA), calculated using DTI fit to $b_{450}:b=100,\mathrm{and}\;450\;s/mm^{2}$ compared to (B) and (F) $b_{900}:b=100,\mathrm{and}\;900\;s/mm^{2}$, (C) and (G) $b_{1200}:b=100,\mathrm{and}\;1200\;s/mm^{2}$, and (D) and (H) $b_{1350}:b=100,\mathrm{and}\;1350\;s/mm^{2}$. Mean difference ± 1.96 SD is given by solid and dashed black lines, respectively (N = 10 subjects).

The mean MD and FA values obtained from DTI fitting of four subsets of data with different maximum b‐values (b = 100 and 450 $s/mm^{2}$, b = 100 and 900 $s/mm^{2}$, b = 100 and 1200 $s/mm^{2}$, b = 100 and 1350 $s/mm^{2}$) for 10 subjects are shown as boxplots in Figure 2. The average MD value reduces from $(1.58\pm 0.04)\times 10^{-3}{\mathrm{mm}}^{2}/s$ at $b_{\max}=450s/mm^{2}$ to $(1.44\pm 0.04)\times 10^{-3}{\mathrm{mm}}^{2}/s$ at $b_{\max}=1350s/mm^{2}$ (Table 1), and the difference is statistically significant (p<0.05) (Table 2). While the mean FA values are almost unchanged at different $b_{\max}$ values (0.31±0.01) (Tables 1 and 2).

**TABLE 1:** Mean ± standard deviation of mean diffusivity (MD) and fractional anisotropy (FA) for DTI fit to different $b_{\max}$ ($b_{\max}=450\;s/mm^{2}$ [b = 100 and 450 $s/mm^{2}$], $b_{\max}=900\;s/mm^{2}$ [b = 100 and 900 $s/mm^{2}$], $b_{\max}=1200\;s/mm^{2}$ [b = 100 and 1200 $s/mm^{2}$], $b_{\max}=1350\;s/mm^{2}$ [b = 100 and 1350 $s/mm^{2}$]) inside a left ventricle mask and then averaged over volunteers. (* shows the statistical significance between the parameters obtained with a specific $b_{\max}$ compared to the one from $b_{\max}=450\;s/mm^{2}$).

$b_{\max}[s/mm^{2}]$	MD [$\times 10^{-3}mm^{2}/s$]	FA
450	1.58 ± 0.04	0.30 ± 0.01
900	1.51 ± 0.04 *	0.31 ± 0.01
1200	1.46 ± 0.04 *	0.31 ± 0.01
1350	1.44 ± 0.04 *	0.31 ± 0.01

**TABLE 2:** Mean difference ± standard deviation between global mean values of MD, FA calculated using DTI fit to $b_{450}:b=100,\;\mathrm{and}\;450\;s/mm^{2}$ compared to $b_{900}:b=100,\mathrm{and}\;900\;s/mm^{2}$, $b_{1200}:b=100,\mathrm{and}\;1200\;s/mm^{2}$, and $b_{1350}:b=100,\mathrm{and}\;1350\;s/mm^{2}$ inside a left ventricle mask.

MD($b_{900}$) − MD($b_{450}$) $\left[\times 10^{-3}mm^{2}/s\right]$	0.07 ± 0.02 (p = 2e−7) (Figure 2B)
MD($b_{1200}$) − MD($b_{450}$) $\left[\times 10^{-3}mm^{2}/s\right]$	0.12 ± 0.01 (p = 5e−10) (Figure 2C)
MD($b_{1350}$) − MD($b_{450}$) $\left[\times 10^{-3}mm^{2}/s\right]$	0.14 ± 0.01 (p = 2e−10) (Figure 2D)
FA($b_{900}$) − FA($b_{450}$)	−0.001 ± 0.005 (p = 0.37) (Figure 2F)
FA($b_{1200}$) − FA($b_{450}$)	−0.005 ± 0.007 (p = 0.03) (Figure 2G)
FA($b_{1350}$) − FA($b_{450}$)	−0.005 ± 0.008 (p = 0.06) (Figure 2H)

**FIGURE 3:**
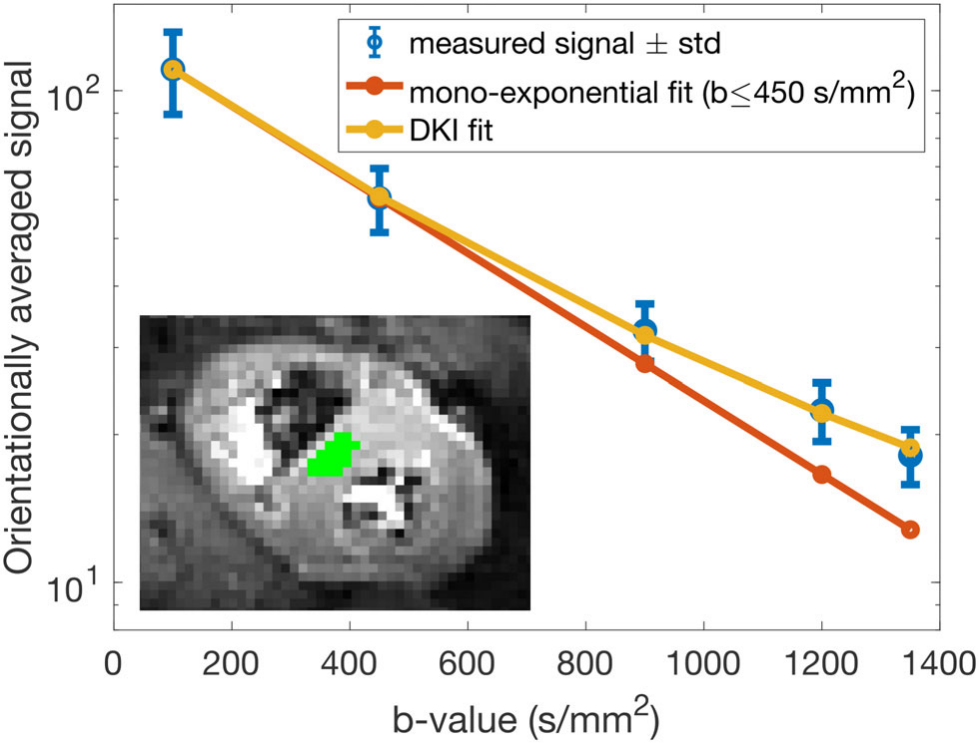
Semi‐log plot of the average signal over the region of interest (ROI), highlighted as green, for each b‐value. The blue dots and the error bars show the mean and standard deviation of the measured signal in the ROI, the red curve shows the mono‐exponential fit to the average signal from $b\leq 450\;s/mm^{2}$, and the yellow curve shows the one‐dimensional DKI fit. The measured signal deviates from the mono‐exponential decay by increasing the b‐value above 450 $s/mm^{2}$.

Figure 3 shows the average signal attenuation versus b‐value in the ROI highlighted as green for a randomly chosen subject. The blue dots and error bars indicate the mean and standard deviation of the measured signal over the ROI at each b‐value, while the mono‐exponential fit to the averaged signal from $b\leq 450s/mm^{2}$ is shown in red and the DKI fit in yellow. Note that here we use a one‐dimensional mono‐exponential fit (red curve) and one‐dimensional DKI as described in Equation 1 (yellow curve). At lower b‐values, $b\leq 450\;s/mm^{2}$, the signal from the mono‐exponential fit and the measured signal are indistinguishable, as expected from the theory. However, the measured signal clearly deviates from the mono‐exponential decay for $b>450\;s/mm^{2}$. The relative difference between the mono‐exponential fit and the measured signal generally increases with b‐value, corresponding to the divergent signal attenuation curves, which is a hallmark of non‐Gaussian diffusion in the tissue.

**FIGURE 4:**
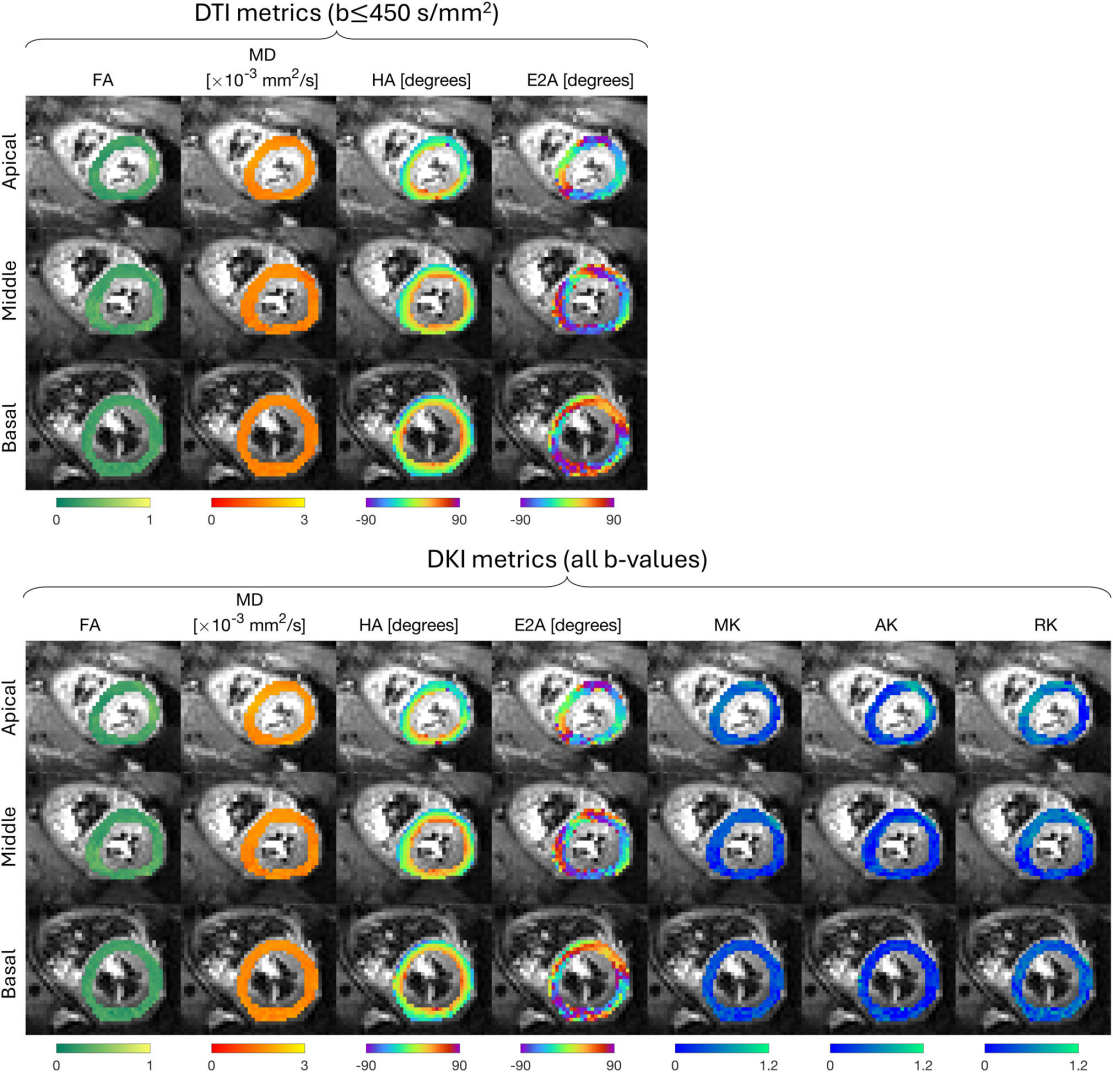
Examples of mean diffusivity (MD), fractional anisotropy (FA), helix angle (HA) and secondary eigenvector angle (E2A) calculated using $b=100\;\mathrm{and}\;450\;s/mm^{2}$ for DTI and FA, MD, HA, E2A, mean, axial, and radial kurtosis (MK, AK, and RK) calculated using all b‐values for DKI.

Representative FA, MD, HA, and E2A maps from DTI and FA, MD, HA, E2A, MK, AK, and RK from DKI fitting for apical, middle, and basal slices are shown in Figure 4.

**FIGURE 5:**
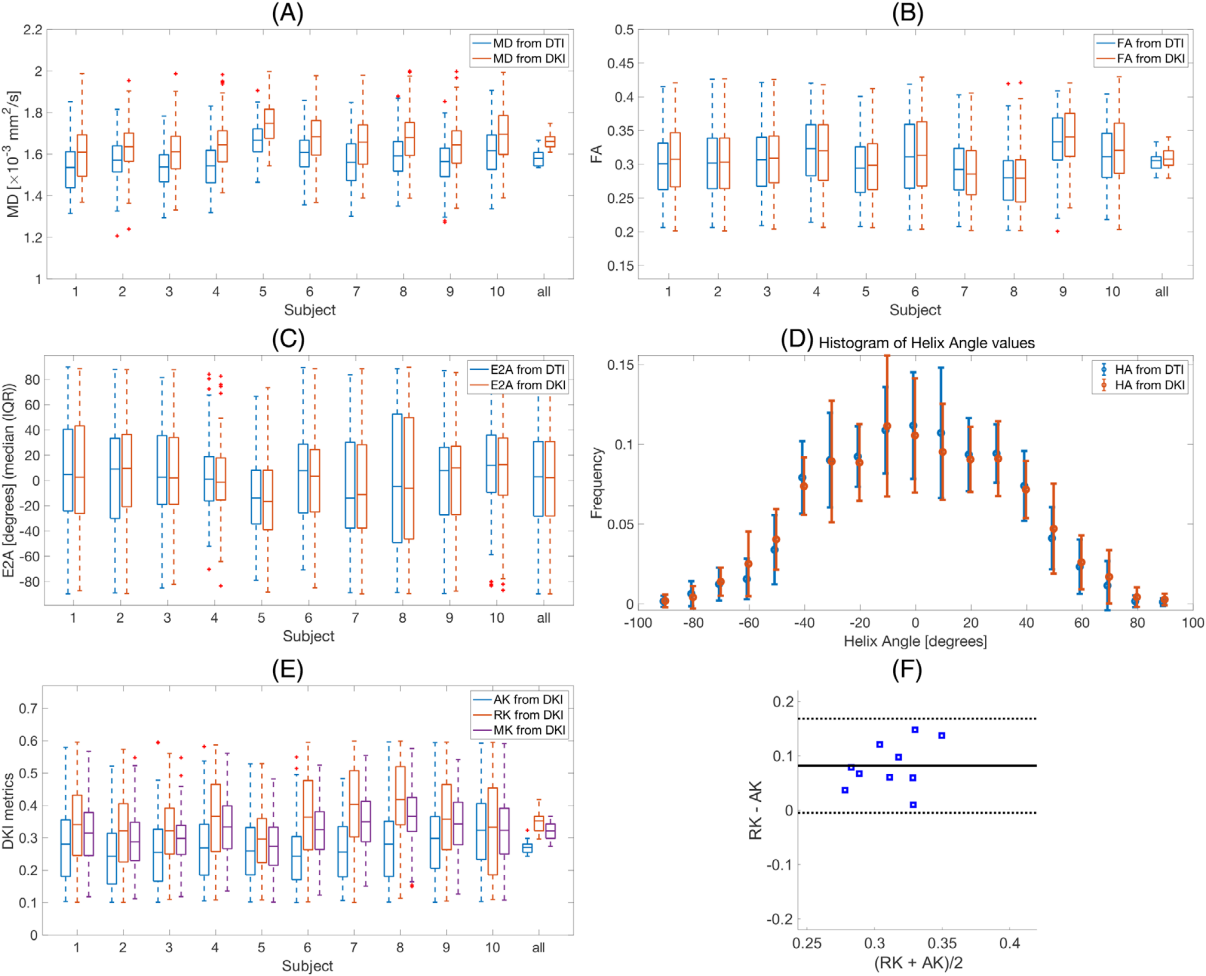
Box plot of (A) mean diffusivity (MD), (B) fractional anisotropy (FA), (C) median and interquartile range (IQR) for secondary eigenvector angle (E2A), (D) histogram of helix angles (HA) from DTI and DKI, and (E) mean kurtosis (MK), axial kurtosis (AK), and radial kurtosis from DKI over left ventricular mask for all 10 subjects (“all” represents the distribution of the mean values in subjects 1 to 10). On each box, the central mark indicates the mean, except (C), which shows the median, and the bottom and top edges of the box indicate the 25th and 75th percentiles, respectively. The whiskers extend to the most extreme data points not considered outliers, and the outliers are plotted individually using the “+” symbol. (F) Bland–Altman plot, comparing axial kurtosis (AK) and radial kurtosis (RK) from DKI. Mean difference ± 1.96 SD are given by solid and dashed black lines, respectively (N = 10).

The mean and standard deviation of MD, FA, MK, AK, RK, the median and interquartile range (IQR) for E2A, and a histogram of HA for all 10 subjects are shown in Figure 5. The average values of the DTI and DKI metrics over 10 subjects are presented in Table 3 (DTI: MD = (1.58 ± 0.04) $\times 10^{-3}{\mathrm{mm}}^{2}/s$, FA = 0.30 ± 0.01, and E2A = 3 (−28 31) degrees. DKI: MD = (1.66 ± 0.04) $\times 10^{-3}mm^{2}/s$, FA = 0.31 ± 0.02, E2A = 2 (−28 31) degrees, MK = 0.32 ± 0.03, AK = 0.27 ± 0.02, and RK = 0.35 ± 0.04). It was found that the radial kurtosis (RK) was slightly higher than axial kurtosis (AK) (RK=0.35±0.06 vs. AK=0.27±0.05) and the difference is statistically significant ($\mathrm{RK}-\mathrm{AK}=0.08\pm 0.04(p=2\times 10^{-4})$) (Table 3 and Figure 5F).

**TABLE 3:** Mean ± standard deviation of mean diffusivity (MD) and fractional anisotropy (FA) and median (interquartile range [25%−75%]) secondary eigenvector angle (E2A) for DTI (b = 100 and 450 $s/mm^{2}$) and MD, FA, E2A, mean kurtosis (MK), axial kurtosis (AK), and radial kurtosis (RK) for DKI (all b‐values) inside a left ventricle mask and then averaged over ten volunteers.

DTI	MD [$\times 10^{-3}mm^{2}/s$]	FA	E2A (degrees) (median [IQR])			
	1.58 ± 0.04	0.30 ± 0.01	3 (−28 31)			

DKI	MD [$\times 10^{-3}mm^{2}/s$]	FA	E2A (degrees) (median [IQR])	MK	AK	RK
	1.66 ± 0.04	0.31 ± 0.02	2 (−28 31)	0.32 ± 0.03	0.27 ± 0.02	0.35 ± 0.04

**FIGURE 6:**
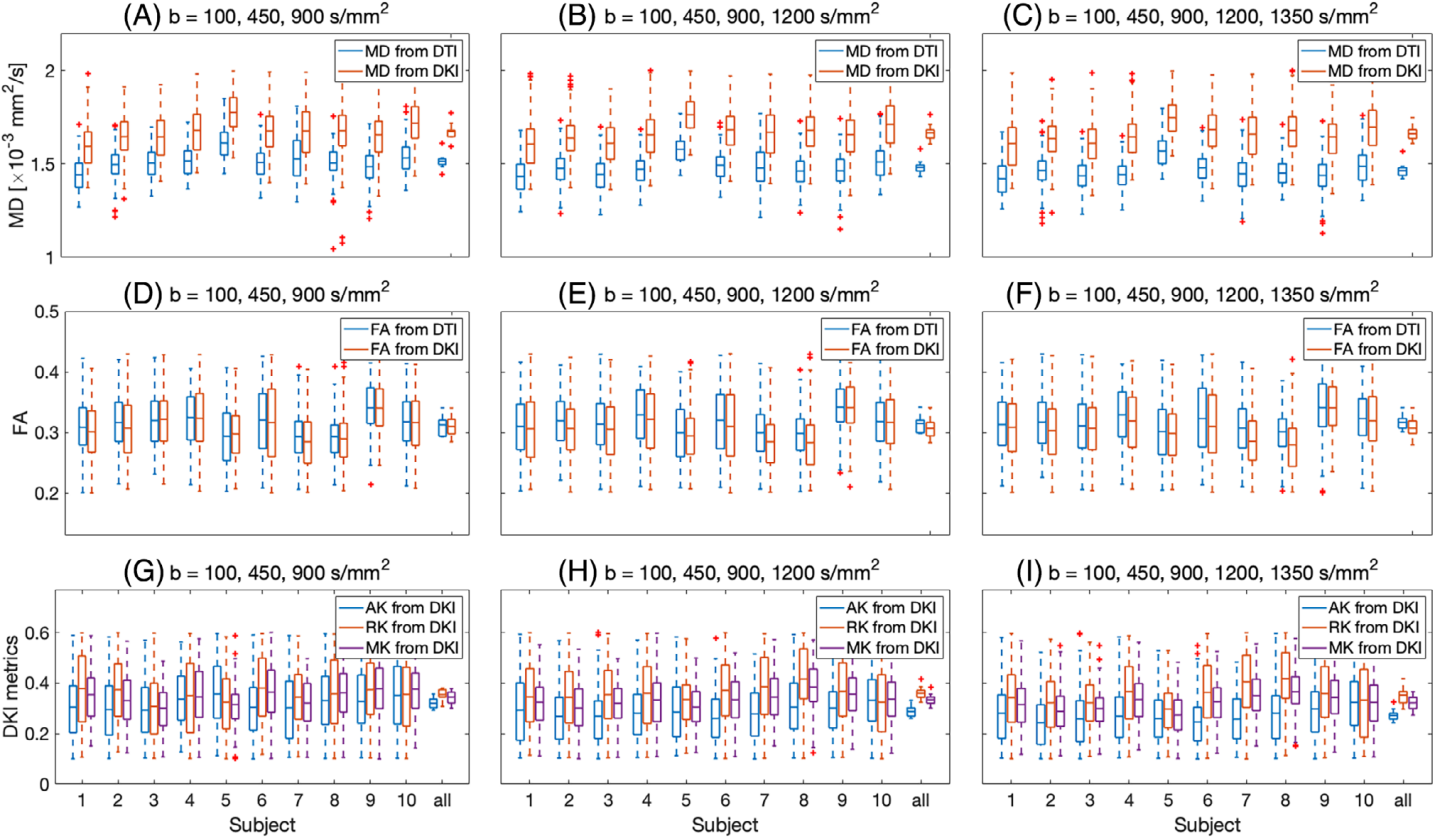
Box plot of (A–C) mean diffusivity (MD) and (D–F) fractional anisotropy (FA) from DTI and DKI and (G–I) mean kurtosis (MK), axial kurtosis (AK), and radial kurtosis from DKI over left ventricular mask for all 10 subjects (“all” represents the distribution of the mean values in subjects 1 to 10). Each column shows the parameters estimated using a subset of b‐values. On each box, the central mark indicates the mean, and the bottom and top edges of the box indicate the 25th and 75th percentiles, respectively. The whiskers extend to the most extreme data points not considered outliers, and the outliers are plotted individually using the “+” symbol.

Comparison between two subsets (subset 1: b = 100, 450, 900 $s/mm^{2}$; subset 2: b = 100, 450, 900, 1200 $s/mm^{2}$) and full set: b = 100, 450, 900, 1200, 1350 $s/mm^{2}$ (Figure 6 and Table 4) shows that by increasing the b‐value the DTI‐estimated MD decreases because of the non‐zero kurtosis effect while the MD from DKI does not change since the kurtosis effect is considered in the kurtosis terms (MK, AK, RK). FA from DTI and DKI does not change with increasing the b‐value. MK and AK slightly decrease while RK is almost unchanged by increasing the b‐value.

**TABLE 4:** Mean ± standard deviation of mean diffusivity (MD) and fractional anisotropy (FA) for DTI and MD, FA, mean kurtosis (MK), axial kurtosis (AK), and radial kurtosis (RK) for DKI using different subsets of b‐value (subset 1: b = 100, 450, 900 $s/mm^{2}$; subset 2: b = 100, 450, 900, 1200 $s/mm^{2}$; full set: b = 100, 450, 900, 1200, 1350 $s/mm^{2}$) inside a left ventricle mask and then averaged over volunteers.

DTI	MD [$\times 10^{-3}mm^{2}/s$]	FA			
Subset 1	1.51 ± 0.04	0.31 ± 0.02			
Subset 2	1.48 ± 0.04	0.31 ± 0.01			
Full set	1.46 ± 0.04	0.32 ± 0.01			

DKI	MD [$\times 10^{-3}mm^{2}/s$]	FA	MK	AK	RK
Subset 1	1.67 ± 0.05	0.31 ± 0.02	0.34 ± 0.03	0.32 ± 0.02	0.35 ± 0.02
Subset 2	1.67 ± 0.05	0.31 ± 0.02	0.33 ± 0.02	0.29 ± 0.02	0.36 ± 0.03
Full set	1.66 ± 0.04	0.31 ± 0.02	0.32 ± 0.03	0.27 ± 0.02	0.35 ± 0.04

## 5 DISCUSSION

This work demonstrates the feasibility of performing DKI in the human heart in vivo using much stronger gradients than commonly available in routine clinical settings. To accurately measure diffusional kurtosis, b‐values higher than those typically used in cardiac DTI are necessary to clearly reveal the deviation from monoexponential decay.^7^, ^16^, ^18^ We further investigated the feasibility of cardiac DKI in systems with lower gradient strength, 200 mT/m (TE = 65 ms) similar to Siemens Healthineers, Magnetom Cima.X and 80 mT/m (TE = 91 ms) which is the gradient strength in the commonly used clinical scanners (see Supporting Information, section Cardiac diffusion kurtosis imaging using different gradient strengths, for more details).

In brain studies, maximum b‐values of about 2000 to 3000 $s/mm^{2}$ ^24^ (two to three times higher than b = 1000 $s/mm^{2}$ used for DTI) are recommended to quantify the kurtosis effect. We followed the same rationale: The maximum b‐value used in cardiac DTI in vivo is around 450 $s/mm^{2}$, so the maximum b‐value of 900 to 1350 $s/mm^{2}$ should be sufficiently high to show the deviation of diffusion weighted signal from the mono‐exponential decay.

Sufficient SNR at high b‐value images is essential to accurately estimate DKI metrics.^45^ Glenn et al.^46^ found that the estimated kurtosis parameters are 93% accurate when the SNR is greater than 3 and greater than 98% for SNR greater than 5. For insufficient SNR, the signal intensity approaches the noise floor, resulting in an artificial curvature of the signal decay and biased estimates of the kurtosis metrics.^46^ Tissue diffusion properties determine the rate at which the signal approaches the noise floor. Obtaining sufficient SNR at high b‐values is particularly challenging in the human heart due to motion and the associated need for motion‐compensated gradient waveforms, which in turn require longer echo times, additionally confounded by short $T_{2}$ of the heart tissue.^47^ By using a high‐performance gradient system in this study, a b‐value of 1350 $s/mm^{2}$ with a minimum TE = 61 ms for the chosen imaging parameters and optimized waveforms was feasible, which significantly improved the SNR efficiency of in vivo cardiac diffusion kurtosis imaging. The achieved sequence timings were comparable to standard cardiac DTI acquisitions at more than three times (>3×) higher b‐values. It provides SNR of 7±2 over the left ventricle for b = 1350 $s/mm^{2}$ which is high enough to avoid diffusion weighted signal falling below the noise floor.^46^ Notably, the same b‐value on clinical routine systems with $G_{\max}$ = 80 mT/m would need an echo time of at least 91 ms (see Supporting Information, Figure S1) (Teh et al.^16^ used TE = 118 ms to enable tensor‐valued diffusion encoding at b‐value of 1500 $s/mm^{2}$). The approximately 30 ms shorter echo time improves the SNR nearly two‐fold due to the short $T_{2}$ of cardiac tissue: Based on the reported $T_{2}$ of around 46 ms^48^ the SNR increase is exp(−61/46)/exp(−90/46)≈1.88 (also see Supporting Information, Table S1).

We found that by increasing b‐value, the MD values from DTI fit decreased (MD = (1.58 ± 0.04) $\times 10^{-3}mm^{2}/s$ at $b_{\max}=450s/mm^{2}$ and MD = (1.44 ± 0.04) $\times 10^{-3}mm^{2}/s$ at $b_{\max}=1350s/mm^{2}$) where this difference is statistically significant (p=2e−10) (Figures 2 and 6, Tables 1, 2, and 4). This reduction in MD is due to the kurtosis effect that is more pronounced at higher b‐values. One may argue that the noise in the data might have a similar effect, however the SNR at the highest b‐value is 7±2 and also the real part of the signal (instead of magnitude) is used in this work to avoid the bias in the signal due to rician noise,^37^ therefore the reduction in MD should be due to the kurtosis effect. The estimated HA and E2A from DTI and DKI are very similar (Figure 5 and Table 3), which is expected since increasing the b‐value does not affect the directional information considerably.^17^

To the best of our knowledge, this is the first report of three‐dimensional diffusion kurtosis in the human heart in vivo. We found that radial kurtosis (RK=0.35±0.04) was slightly larger than axial kurtosis (AK=0.27±0.02) and the difference was statistically significant (RK − AK = 0.08 ± 0.04, p=2e−4, Table 3 and Figure 5D). Teh et al.^16^ previously reported total kurtosis of 0.33±0.09 for in vivo human heart on a standard clinical MR scanner, which aligns with our results on ultra‐strong gradient system (MK = 0.32 ± 0.03); however, they did not quantify the three‐dimensional kurtosis metrics such as AK and RK. It is known that there are more restrictions perpendicular to the cardiomyocyte orientations than parallel orientations, and a previous study on ex vivo rat^7^ and pig hearts^23^ showed higher RK compared to AK. Mcclymont et al.^7^ reported a kurtosis value of 0.13 ± 0.02, 0.45 ± 0.04 and 0.55 ± 0.03 along first, second and third eigenvector. We observe the trend of higher RK compared to AK in our work; however, the kurtosis values in the in vivo human heart are slightly different than the values reported ex vivo. The reason is that the tissue preparation/fixation may affect the cell size and cause shrinkage, which results in a higher kurtosis effect along the perpendicular direction. In addition, the difference between species, that is, rat heart versus human heart, may contribute to this difference.

The magnitude of kurtosis observed in the in vivo human heart is limited due to the microstructure of the cardiac tissue. We used a simple biophysical model to simulate the signal from cardiac tissue, and the estimated kurtosis values in the simulated signal are close to the values we obtained in vivo (see Supporting Information, section Simulation, for more details). We further investigated the effect of diffusion encoding time on the kurtosis values and we found that by increasing the effective diffusion time, the amount of mean and radial kurtosis (MK and RK) slightly increase (Table S3), however, the increase in diffusion encoding time, prolonges the echo time and therefore reduces the SNR.

The study was conducted on a limited cohort of healthy volunteers; the extrapolation of this technique to patient populations with specific cardiac pathologies is the topic of our future work. Diffusion kurtosis metrics are sensitive to tissue microstructure but are not specific to particular microstructural features (e.g., intracellular and extracellular space).

One possible clinically feasible protocol could include b‐values of 100, 450, and 900 $s/mm^{2}$ with 3, 30, and 30 diffusion encoding directions and 12, 6, and 6 repetitions, respectively. This reduced protocol has a total scan time of ∼20 min, making it more suitable for clinical settings. The results for such protocol are shown in the first column of Figure 6 and as subset 1 in Table 4. It can clearly be seen that the estimated parameters from the reduced protocol (subset 1) are similar to the ones obtained from the full protocol (full set), with only minor differences in MK and AK. Further investigation into the optimal combination of b‐values, diffusion directions, and repetitions, while maintaining the integrity of the diffusion metrics, will be subject to future work.

The primary purpose of our work was to establish the feasibility of DKI in human hearts in vivo using ultra‐strong gradients, and highly relevant with the advent of the latest generation of clinical MR scanners with 200 mT/m gradient systems (such as the Siemens Healthineers, Magnetom Cima.X). This opens the field for novel investigation and paves the way for the exploration of cardiac DKI in clinical studies.

## 6 CONCLUSION

In this work, we demonstrated the feasibility of three‐dimensional DKI in the human heart in vivo. This was facilitated using strong gradients (300 mT/m) that could provide high b‐values ($b_{\max}=1350s/mm^{2}$) for a second‐order motion‐compensated waveform at a TE = 61 ms. Radial kurtosis (RK) was observed to be slightly larger than axial kurtosis (AK), as expected, and the difference was statistically significant. The in vivo measurement of radial, axial, and mean kurtosis provides the potential for characterizing the myocardial microstructure, which may be useful in some cardiac diseases.

## CONFLICT OF INTEREST STATEMENT

FF was employed by the company Siemens Healthcare Ltd. FS declares ownership interests in Random Walk Imaging, which holds patents related to the methodology. The remaining authors declare that the research was conducted in the absence of any commercial or financial relationships that could be construed as a potential conflict of interest.

## Supporting information


**Data S1.** Supporting Information.

## Data Availability

The data used in this manuscript will be provided upon request.
