# Supplementary material for: Cardiac diffusion kurtosis imaging in the human heart in vivo using 300 mT/m gradients
Source: Magn Reson Med. 2025 Jul 3;94(5):2100–12. doi: 10.1002/mrm.30626 (PMC12393209; doi:10.1002/mrm.30626)
Supplement: Supplementary file 1 — Data S1. Supporting Information. [file MRM-94-2100-s001.pdf]

Supporting information

Cardiac diffusion kurtosis imaging using different gradient strengths

Cardiac diffusion weighted images were acquired from three healthy subjects using maximum gradient strength of 300, 200, and 80 mT/m corresponding to the Connectom, Cima.X, and Prisma (a commonly used clinical MRI scanner) Siemens MRI scanners, respectively. All the acquisitions were performed on the Connectom MR system (G_{max} = 300mT/m) and the gradient strength was adjusted for each scenario by increasing the timing of the diffusion gradients. Figure S1 shows the numerically optimized second-order motion compensated waveform for b_{max} = 1350 s/mm^2, (A) G_{max} = 285.4 mT/m, maximum slew rate of 76.2 T/m/s, TE = 61 ms (B) G_{max} = 193.2 mT/m, maximum slew rate of 77.3 T/m/s, TE = 65 ms (C) G_{max} = 76.9 mT/m, maximum slew rate of 76.9 T/m/s, TE = 91 ms (maximum slew rate is lower than the technical limit to reduce PNS and avoid cardiac stimulation). Each full data set was comprised of 5 b-values [b = 100, 450, 900, 1200, 1350 s/mm^2] in 30 directions per shell with 2 repetitions, except for the lowest b-value which only had 3 directions and 4 repeats. By reducing the gradient strength from 300 mT/m to 80 mT/m, a longer TE (61 vs. 91 ms) is needed to provide the same maximum b-value (1350 s/mm^2). The change in echo time is not considerable between G_{max} = 300 and 200 mT/m (61 vs. 65 ms).

Representative diffusion weighted images acquired with b = 100, 450, 900, 1200, and 1350 s/mm^2 and G_{max} = 300, 200, and 80 mT/m are shown in Figure S2 for a single diffusion direction. The change in image quality between G_{max} = 300 and 80 mT/m is considerable while the quality of images acquired at G_{max} = 300 and 200 mT/m is very similar. The average SNR values for different G_{max} and b-values are reported in Table S1 . By increasing the gradient strength the SNR considerable, for example it changes from 19 ± 4 at G_{max} = 80 mT/m to 43 ± 10 at G_{max} = 300 mT/m for b = 100 s/mm^2.

Representative fractional anisotropy (FA), mean diffusivity (MD), helix angle (HA), secondary eigenvector angle (E2A), mean kurtosis (MK), axial kurtosis (AK), and radial kurtosis (RK) for different gradient strengths G_{max} = 300, 200, 80 mT/m are shown in Figure S3 . Mean ± standard deviation of MD, FA, MK, AK, and RK using different gradient strengths (G_{max} = 300, 200, 80 mT/m) are given in Table S2 . By reducing the gradient strength from 300 to 80 mT/m, the mean MD value increases from 1.71 to 1.74 $\times 10^{-3} mm^2/s$, FA, MK, AK increase, and RK decreases. These changes could be due to the longer echo time necessary at the lower gradient strength (80 mT/m) and therefore reduced SNR. The results are similar for gradient strengths of 300 and 200 mT/m.

Table S1 : Calculated signal-to-noise-ratios (SNRs) for different G_{max} = 300, 200, 80 mT/m and different b-values b = 100, 450, 900, 1200, 1350 s/mm^2.

SNR	b = 100	b = 450	b = 900	b = 1200	b = 1350
G_{max} = 300 mT/m	43 ± 10	26 ± 6	14 ± 4	9 ± 3	8 ± 2
G_{max} = 200 mT/m	38 ± 6	23 ± 5	13 ± 3	8 ± 2	7 ± 2
G_{max} = 80 mT/m	19 ± 4	11 ± 2	6 ± 1	4 ± 1	3 ± 1

Table S2 : Mean ± standard deviation of mean diffusivity (MD), fractional anisotropy (FA), mean kurtosis (MK), axial kurtosis (AK), and radial kurtosis (RK) using different gradient strength (G_{max} = 300, 200, 80 mT/m) inside a left ventricle mask and then averaged over volunteers.

	MD [$\times 10^{-3}$ mm^2/s]	FA	MK	AK	RK
G_{max} = 300 mT/m	1.71 ± 0.07	0.33 ± 0.01	0.34 ± 0.02	0.30 ± 0.02	0.35 ± 0.02
G_{max} = 200 mT/m	1.70 ± 0.09	0.34 ± 0.01	0.36 ± 0.04	0.33 ± 0.04	0.35 ± 0.03
G_{max} = 80 mT/m	1.74 ± 0.01	0.36 ± 0.01	0.36 ± 0.03	0.35 ± 0.02	0.32 ± 0.05

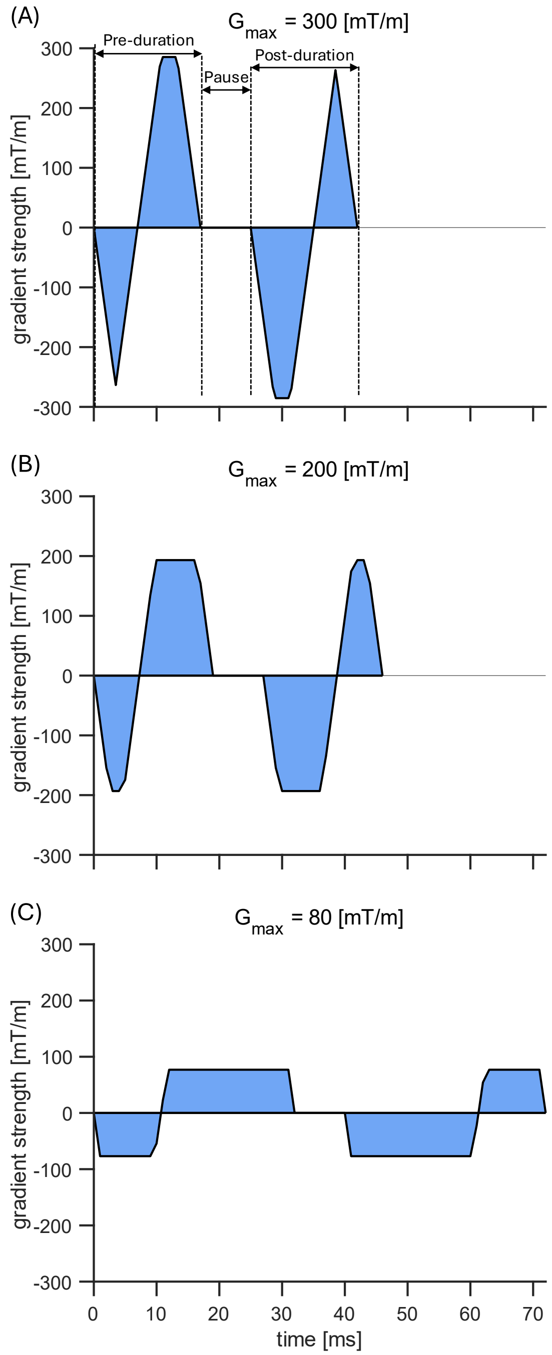

Figure S1 : Numerically optimized second-order motion compensated waveform for $b_{max} = 1350\,s/mm^2$, (A) $G_{max} = 300\,mT/m$, TE = 61 ms (B) $G_{max} = 200\,mT/m$, TE = 65 ms (C) $G_{max} = 80\,mT/m$, TE = 91 ms.

Simulations

Cardiomyocytes are 17 to 25 μm in diameter (Tracy and Sander, 2011) therefore the amount of restriction is limited compared to the brain white matter where the axons have a radius of a few micrometers. To support this statement, we simulated the signal from a simple multi-compartment model including a cylinder representative of cardiomyocyte and a zeppelin for the extra-cellular space. Therefore, the

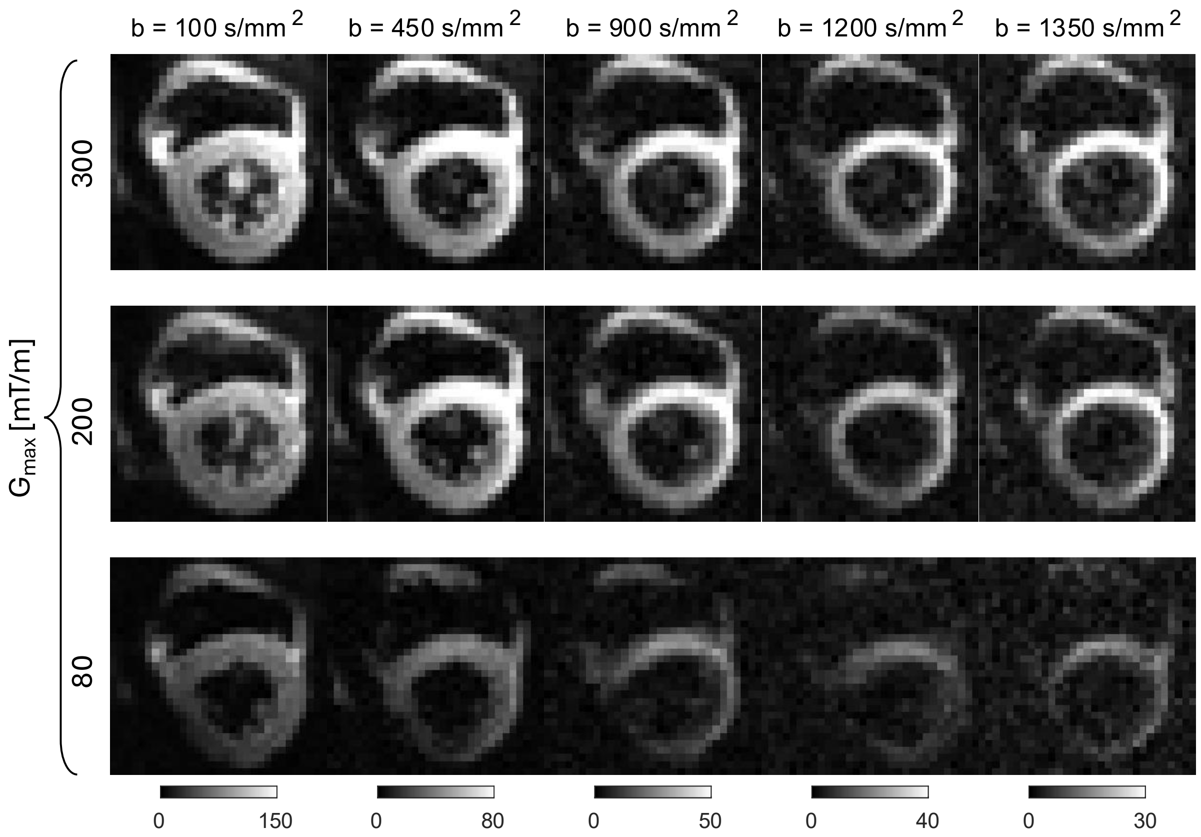

Figure S2 : Representative diffusion weighted images acquired with b = 100, 450, 900, 1200, and 1350 s/mm^2 and G_{max} = 300, 200, and 80 mT/m for a single diffusion direction.

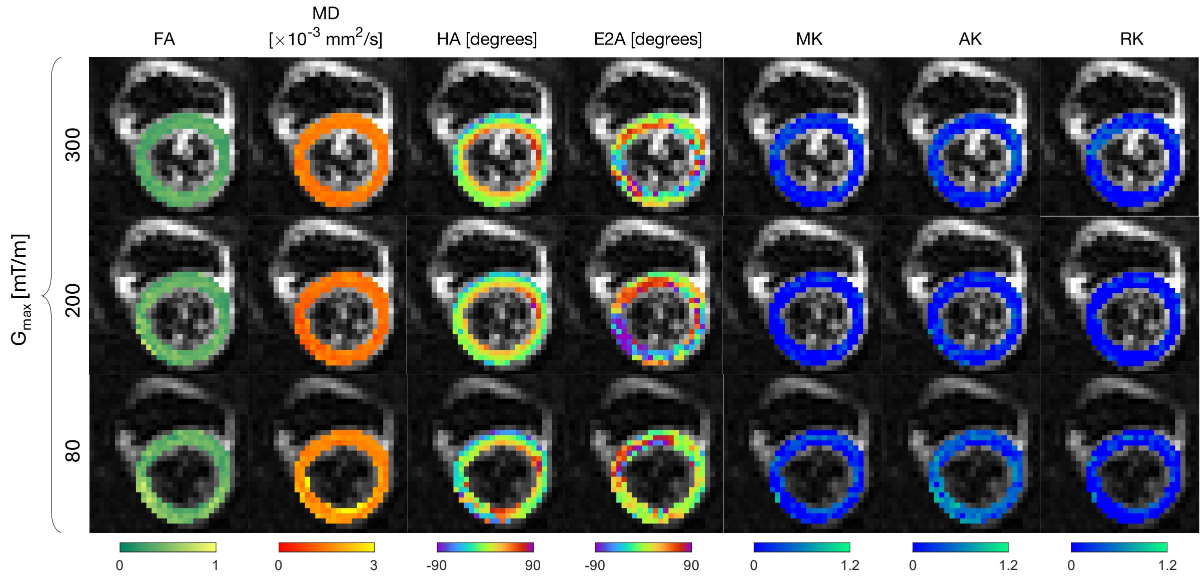

Figure S3 : Representative fractional anisotropy (FA), mean diffusivity (MD), helix angle (HA), secondary eigenvector angle (E2A), mean, axial and radial kurtosis (MK, AK, and RK) for different gradient strengths G_{max} = 300 200, 80 mT/m.

simulated diffusion-weighted signal is described as a sum of two components:

$$S = f_{cylinder} S_{cylinder}(R_{cylinder}, D^{||}_{cylinder}) + f_{zeppelin} S_{zeppelin}(D^{||}_{zeppelin}, D^{\perp}_{zeppelin}) \tag{R1}$$

where $f_{cylinder}$, $f_{zeppelin}$, $S_{cylinder}$ and $S_{zeppelin}$ are the signal fractions and the diffusion weighted signal from the cardiomyocyte and extra-cellular components ($f_{cylinder} + f_{zeppelin} = 1$). While $R_{cylinder}$, $D^{||}_{cylinder}$, $D^{||}_{zeppelin}$, and $D^{\perp}_{zeppelin}$ are the cylinder radius and diffusivity and zeppelin diffusivities, respectively (Afzali et al., 2021).

The following parameters from the literature were used for the simulation (Farzi et al., 2021; Poole-Wilson, 1995; Tracy and Sander, 2011), $[f_{cylinder}, f_{zeppelin}, R_{cylinder}, D^{||}_{cylinder}, D^{||}_{zeppelin}, D^{\perp}_{zeppelin}] = [0.65, 0.35, 9\,\mu m, 3\,\mu m^2/ms, 1.5\,\mu m^2/ms, 1\,\mu m^2/ms]$. The timing of the diffusion encoding waveform is 17, 8, and 17 ms for the pre-, pause- and post-duration, respectively (Figure **??**). Fitting the kurtosis tensor leads to mean kurtosis (MK) = 0.31, axial kurtosis (AK) = 0.26 and radial kurtosis (RK) = 0.33 which is close to the values we obtained in the in vivo analysis. By increasing the effective diffusion time, mean kurtosis and radial kurtosis increase slightly (Table S3).

Table S3 : Calculated mean kurtosis (MK), axial and radial kurtosis (AK and RK) from simulated signal for different effective diffusion times.

pre-pause-post duration [ms]	MK	AK	RK
17-8-17	0.31	0.26	0.33
20-8-20	0.31	0.26	0.34
25-8-25	0.32	0.26	0.36
30-8-30	0.33	0.26	0.37
35-8-35	0.33	0.26	0.37

References

Afzali, M., Nilsson, M., Palombo, M., Jones, D.K.. Spheriously? the challenges of estimating sphere radius non-invasively in the human brain from diffusion MRI. NeuroImage 2021;237:118183.

Farzi, M., Mcclymont, D., Whittington, H., Zdora, M.C., Khazin, L., Lygate, C.A., Rau, C., Dall'Armellina, E., Teh, I., Schneider, J.E.. Assessing myocardial microstructure with biophysical models of diffusion mri. IEEE transactions on medical imaging 2021;40(12):3775–3786.

Poole-Wilson, P.A.. The dimensions of human cardiac myocytes; confusion caused by methodology and pathology. Journal of molecular and cellular cardiology 1995;27(3):863–865.

Tracy, R.E., Sander, G.E.. Histologically measured cardiomyocyte hypertrophy correlates with body height as strongly as with body mass index. Cardiology research and practice 2011;2011(1):658958.
